# Dual Dialysis for Post-bilateral Orthotopic Lung Transplantation Hyperammonemia

**DOI:** 10.7759/cureus.63607

**Published:** 2024-07-01

**Authors:** Aniruddha Bhattacharyya, Girma M Ayele, Samrawit W Zinabu, Rediet Tefera Atalay, Ahmad Mohammed, Mahlet Siraga, Lucia Gao, Bharadwaj Adithya Sateesh, Huda Gasmelseed, Miriam B Michael

**Affiliations:** 1 Internal Medicine, University of Maryland, Baltimore, USA; 2 Internal Medicine, Howard University Hospital, Washington, D.C., USA; 3 Medicine, University of Maryland, Baltimore, USA; 4 Medicine, American University of Antigua, St John's, ATG

**Keywords:** post-lung transplant complications, continuous renal replacement therapy (crrt), encephalopathy, non-cirrhotic hyperammonemia, dialyisis

## Abstract

Hyperammonemia is a metabolic disorder characterized by supraphysiologic ammonia (NH_3_) concentrations in the blood. Although usually seen in adults with liver disease, hyperammonemia is a notable complication in 4.1% of lung transplants. It is associated with cerebral edema and neurological dysfunction and carries up to 75% mortality in critically ill patients. Opportunistic infections caused by *Mycoplasma *and *Ureaplasma* species have been implicated as the cause of this metabolic disturbance. Literature in neonates has shown that renal replacement therapy (RRT) is the best choice for treating patients with neurologic manifestations of hyperammonemia, in cases of NH3 clearance than continuous renal replacement therapy (CRRT). In contrast, continuous venovenous hemodialysis (CVVHD) is usually better tolerated for patients with hemodynamic instability for NH_3_ clearance. NH_3_ is a small molecule whose clearance mirrors urea in dialysis. Even though RRT can be a treatment modality for hyperammonemia in adults and neonates, there is very little literature on adults. We present a unique case demonstrating improvement in neurologic manifestations of hyperammonemia by using both IHD and CVVHD in an adult patient.

## Introduction

Hyperammonemia syndrome (HS) following lung transplantation is a well-recognized complication associated with significant morbidity and mortality. While the overall incidence of HS is elevated in lung transplant recipients compared to other patient populations [1], *Ureaplasma *infection appears to be a particularly potent risk factor, with a strikingly high incidence of HS (41.67%) observed in this subgroup. This starkly contrasts the mere 2.84% incidence observed in *Ureaplasma*-negative recipients [2]. Moreover, *Ureaplasma*-associated HS carries a substantially higher mortality rate (27.27%) compared to HS arising from other etiologies (5.24%) [2]. These findings underscore the critical role of *Ureaplasma* infection in the pathogenesis of post-transplant HS and highlight the need for targeted preventative and therapeutic strategies in this high-risk population.

Literature in neonates has shown that renal replacement therapy (RRT) is the best choice for treating patients with neurologic manifestations of hyperammonemia. In cases of hyperammonemia in infants, intermittent hemodialysis (IHD) demonstrated faster ammonia (NH_3_) clearance than continuous renal replacement therapy (CRRT). In contrast, continuous venovenous hemodialysis (CVVHD) is usually better tolerated for patients with hemodynamic instability for NH_3_ clearance. NH_3 _is a small molecule whose clearance mirrors urea in dialysis. Even though renal replacement therapy can be a treatment modality for hyperammonemia in adults and neonates, there is very little literature on adults [3].

## Case presentation

A 64-year-old male was hospitalized for a scheduled bilateral orthotopic lung transplantation (BOLT). His past medical history was significant for chronic obstructive pulmonary disease (COPD), requiring baseline supplemental oxygen of 2-4 L, and for a pulmonary abscess complicated by recurrent left-sided pneumothorax treated with right upper lobe posterior segmentectomy and left wedge resection with talc pleurodesis two months before the initial presentation. He also had a history of gastroesophageal reflux disease (GERD), hyperlipidemia, and chronic osteomyelitis of T4-T5 vertebrae. The patient’s social history is notable for having quit smoking after a 60-pack-year history, occasional cannabis use, and a diagnosis of alcohol use disorder, with a consumption of 21 standard drinks per week. 

The patient received a lung transplant from a cadaveric donor whose cause of death was trauma. His immunosuppression was perioperative induction with basiliximab and methylprednisolone. During the BOLT procedure, he received four units of packed red blood cells, two units of platelets, three units of fresh frozen plasma, four units of cryoprecipitate, and three units of fibrinogen. The patient tolerated the procedure well and remained intubated. He was admitted to the intensive care unit (ICU) for further monitoring.

On the first postop day after successful extubation following BOLT, his initial computed tomography (CT) head and CT angiography of the head and neck were negative for identifiable stroke or acute cerebral edema. A metabolic workup revealed hypernatremia and hyperammonemia with a sodium and NH_3_ concentration of 153 mmol/L and 526 µmol/L, respectively. Subsequent CT scans of his head revealed new cerebral edema. Bronchial fluid obtained after receiving BOLT was tested via polymerase chain reaction assay and detected *Ureaplasma *species' 16S ribosomal RNA sequence. 

**Figure 1:**
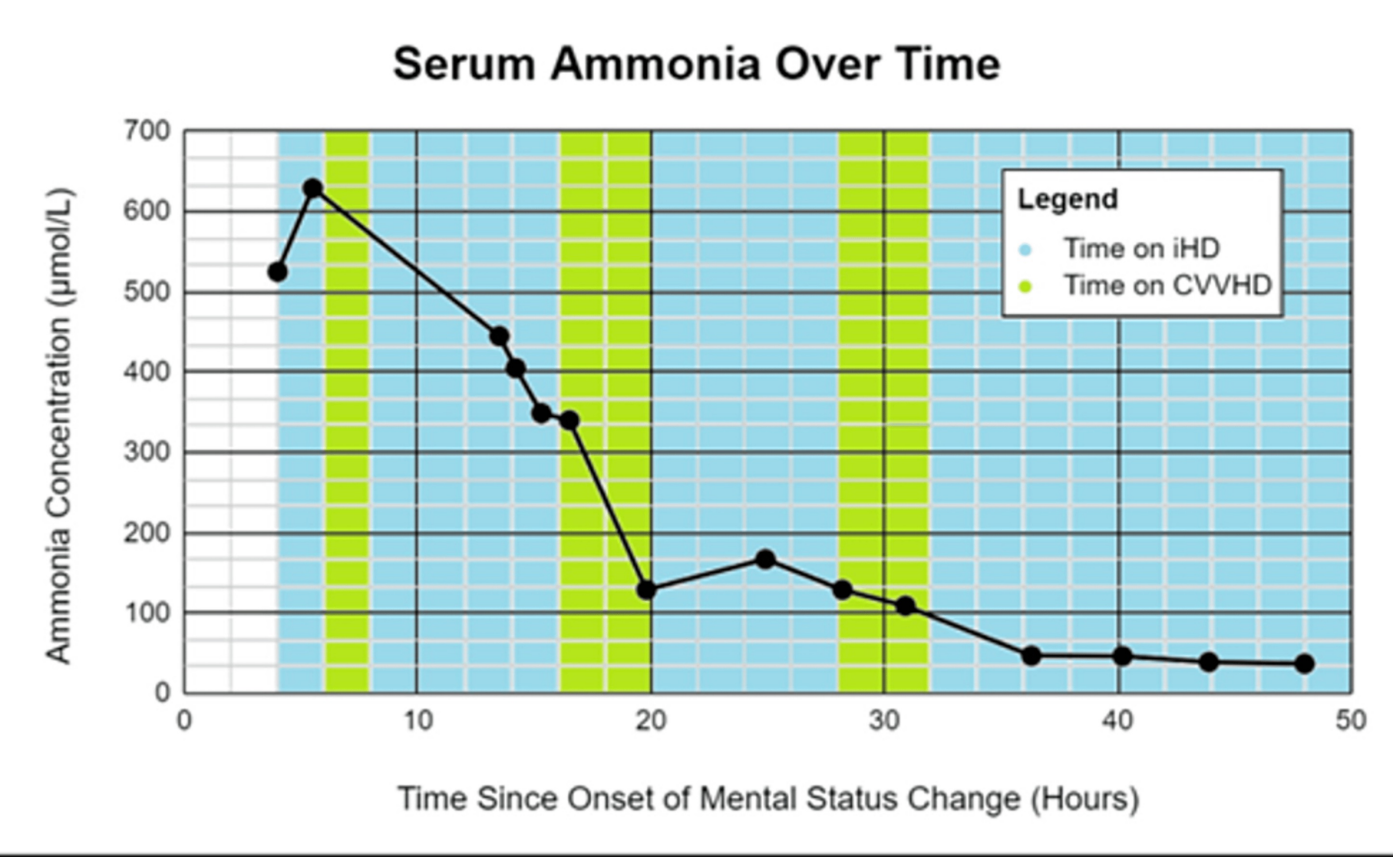
The patient's ammonia concentration change for the 50 hours since the onset of the mental status change. Blue columns indicate the time the patient was on IHD. Green columns indicate the time the patient was on CVVHD IHD: Intermittent hemodialysis; CVVHD: continuous venovenous hemodialysis

Shortly after his hyperammonemia was detected, the patient developed hemodynamic instability that required three pressors for stabilization. While receiving aggressive pressure support, his hyperammonemia was treated with a novel combination of alternating CVVHD and IHD. RRT was performed through a right-sided femoral hemodialysis catheter placed nonemergently with aseptic techniques. The initial prescription for CVVHD involved a blood flow rate (QB) of 300 ml/min and a dialysate flow rate (QD) of 7800 ml/h. For the initial two hours of IHD, the patient had a QB and QD of 200 and 400 ml/min, respectively (Figure 1). The second round of IHD had a QB and QD of 300 and 500 ml/min, respectively, while the third round had a QB and QD of 350 and 600 ml/min, respectively (Figure 1). This unique RRT protocol successfully reduced the patient’s NH_3_ level by 95% over 45 hours. The sodium level was also corrected slowly to 145 over 5-6 days. However, despite the improvements in his hyperammonemia, the patient expired shortly afterward due to opportunistic pneumonia.

## Discussion

Hyperammonemia, a rare complication in lung transplant patients, is associated with a significant mortality rate. In 2020, 2597 lung transplants were performed, with bilateral lung transplants accounting for 78% of procedures. Posttransplant survival trends remained stable, with 89.4% of recipients in 2019 surviving one year, 61.2% in 2015 to five years, and 33.1% in 2010 to 10 years [4]. Although hyperammonemia is a rare complication of lung transplants affecting about 4% of patients, it carries up to 75% mortality. Opportunistic infections caused by *Mycoplasma* and *Ureaplasma *species have been implicated as the causes of this metabolic disturbance [2]. Additionally, hyperammonemia can have many other causes; noncirrhotic hyperammonemia in adults can be due to hematological disorders such as multiple myeloma, acute leukemia, and infections with other urease-producing organisms (*E. coli*, *Klebsiella*, and *Proteus*) [5]. Unmasked urea cycle defects, congenital disorders involving enzymatic defects of the urea cycle, and certain drugs like valproic acid, carbamazepine, salicylates, and sulfadiazine can also cause hyperammonemia [5]. 

Mycoplasmas are the smallest free-living, self-replicating organisms and have been associated with cases of hyperammonemia. They are widespread as parasites of humans, mammals, reptiles, fish, arthropods, and plants. Mycoplasmas are found mainly in the oral cavity, the upper respiratory tract, and the distal parts of the genitourinary tracts of humans [2]. Ureaplasma resides in the urogenital and respiratory tract, using urea to produce adenosine triphosphate (ATP) [2]. *Ureaplasma *is transmitted through sexual contact; it can also be transmitted vertically from mother to offspring [2]. Persons who are immunosuppressed due to congenital antibody deficiencies, organ transplantation, or extremely preterm neonates may be susceptible to disseminated infection. The risk of developing hyperammonemia is higher in lung transplant recipients than in other solid organ recipients. An explanation for this disparity is that the organism is aerophilic, and the lung lacks pulmonary defensive mechanisms compared to the genitourinary system, with the urinary tract microbiome and associated acidic environment as host defenses [1].

The exact pathogenesis of NH_3_ causing central nervous system (CNS) damage is not fully understood. However, it is thought to alter the neurotransmitter system [5]. Hyperammonemia can cause cerebral edema due to the swelling of astrocytes, which are responsible for NH_3_ detoxification in the brain [5]. Acute rise in NH_3_ exceeding 200 μmol/L can rapidly cause severe neurological complications, potentially leading to death [6]. Chronic hyperammonemia results in two major pathological changes: increased inhibitory neurotransmission due to the downregulation of glutamate receptors and increased GABAergic tone due to benzodiazepine receptor overstimulation. NH_3 _also increases the transport of tryptophan, resulting in increased serotonin levels in the brain, which can cause anorexia [5]. 

Hyperammonemia is managed by reducing NH_3_ production and absorption in the gastrointestinal tract or removing NH_3_ from the bloodstream. Preemptive or early use of antibiotics to treat *Ureaplasma* infection reduces NH_3_ production. Some antibiotics that treat *Ureaplasma *infection include fluoroquinolones, tetracyclines, chloramphenicol, and macrolides. Treatment, however, can be hampered by a large amount of antimicrobial resistance, and adverse drug reactions to antibiotics need to be given due attention in transplant patients [7]. Antimicrobial-resistant strains of *Ureaplasma *spp. are a growing concern among neonates and can also potentially threaten immunocompromised patients with hyperammonemia syndrome. Eradication of urease-producing bacteria in the gut with nine of 10 antibiotics, that is, neomycin and metronidazole, has also been suggested to decrease NH_3 _production and prevent its absorption in the gastrointestinal tract. Even with antimicrobial therapy, recovery of some patients is complicated due to the development of resistance [8].

RRT has been successfully employed to directly reduce NH_3_ levels in patients’ blood. Currently, there is disagreement on optimal dialysis starting time; some clinicians recommend initiating dialysis if NH_3_ levels rise more than three times the upper limit of normal, provided that the patient does not also have liver disease [9]. The primary objective is to rapidly decrease NH_3_ levels [10]. Delays between diagnosis and dialysis initiation may contribute to negative outcomes. Prolonged dialysis sessions lasting ≥6 hours, with a blood flow rate of 400 ml/min and a fluid flow rate of 800 ml/min, are more effective in NH_3_ clearance [10]. NH_3_ elimination relies on blood flow and is affected by dialysate flow rate and dialyzer surface area. Under clinically feasible conditions, setting a high dialysate flow rate can extract over 80% of NH_3_. IHD using a dialyzer with a large surface area proves more efficient than CRRT, peritoneal dialysis, or charcoal hemoperfusion.

Case studies have demonstrated the benefits of early initiation of high-dose IHD in adult lung transplant recipients. Besides NH_3_, hemodialysis facilitates the removal of urea and glutamine, which can be considered NH_3_ equivalents and are eliminated depending on flow rates. If IHD is inappropriate due to hemodynamic instability, sustained low-efficiency dialysis or CVVHD should be considered at rates of 250 ml/kg/h and 40-50 ml/kg/h, respectively, with maximized blood flows. High flux daily hemodialysis, alongside other supplementary therapies, should also be taken into consideration [9,10]. Additionally, a retrospective institutional review and systematic analysis conducted in 2017 concerning hyperammonemia in lung transplant recipients revealed mortality rates of 40% with intermittent hemodialysis, 75% with CVVHD, and 100% among patients not undergoing RRT for NH_3_ removal, underscoring the critical role of dialysis in these cases [11]. As the literature continues to grow, further studies are needed to determine the best method to reduce mortality in particular patient populations [11].

## Conclusions

Treatment for hyperammonemia should involve a comprehensive strategy, including discontinuation of medications that hinder the urea cycle, aggressive reduction of ammonia levels through prolonged daily IHD, and overnight slow dialysis, along with early tapering of steroids. This case underscores the critical role of early, high-dose, and frequent HD in managing hyperammonemia.
